# Impact of an educational program and decision tool on choice of maternity hospital: the delivery decisions randomized clinical trial

**DOI:** 10.1186/s12884-022-05087-y

**Published:** 2022-10-10

**Authors:** Ateev Mehrotra, Adam Wolfberg, Neel T. Shah, Avery Plough, Amber Weiseth, Arianna I. Blaine, Katie Noddin, Carter H. Nakamoto, Jessica V. Richard, Dani Bradley

**Affiliations:** 1 Department of Health Care Policy, Harvard Medical School, 180 Longwood Ave, Boston, MA 02115, 617-432-3905 US; 2 Ovia Health, Boston, MA US; 3 Ariadne Labs, Harvard T.H. Chan School of Public Health, Boston, MA US; 4 Department of Obstetrics and Gynecology, Beth Israel Deaconess Medical Center, Boston, MA US

**Keywords:** Cesarean delivery, Patient engagement, Public reporting of quality, Randomized controlled trial

## Abstract

**Background:**

Reducing cesarean rates is a public health priority. To help pregnant people select hospitals with lower cesarean rates, numerous organizations publish publically hospital cesarean rate data. Few pregnant people use these data when deciding where to deliver. We sought to determine whether making cesarean rate data more accessible and understandable increases the likelihood of pregnant people selecting low-cesarean rate hospitals.

**Methods:**

We conducted a 1:1 randomized controlled trial in 2019–2021 among users of a fertility and pregnancy mobile application. Eligible participants were trying to conceive for fewer than five months or were 28–104 days into their pregnancies. Of 189,456 participants approached and enrolled, 120,621 participants met entry criteria and were included in analyses. The intervention group was offered an educational program explaining the importance of hospital cesarean rates and an interactive tool presenting hospital cesarean rates as 1-to-5-star ratings. Control group users were offered an educational program about hospital choice and a hospital choice tool without cesarean rate data. The primary outcome was the star rating of the hospital selected by each patient during pregnancy. Secondary outcomes were the importance of cesarean rates in choosing a hospital and delivery method (post-hoc secondary outcome).

**Results:**

Of 120,621 participants (mean [SD] age, 27.8 [7.9]), 12,284 (10.2%) reported their choice of hospital during pregnancy, with similar reporting rates in the intervention and control groups. Intervention group participants selected hospitals with higher star ratings (2.52 vs 2.16; difference, 0.37 [95% CI, 0.32 to 0.43] *p* < 0.001) and were more likely to believe that the hospitals they chose would impact their chances of having cesarean deliveries (38.5% vs 33.1%, *p* < 0.001) but did not assign higher priority to cesarean delivery rates when choosing their hospitals (76.2% vs 74.3%, *p* = 0.05). There was no difference in self-reported cesarean rates between the intervention and control groups (31.4% vs 31.4%, *p* = 0.98).

**Conclusion:**

People offered an educational program and interactive tool to compare hospital cesarean rates were more likely to use cesarean data in selecting a hospital and selected hospitals with lower cesarean rates but were not less likely to have a cesarean.

**Clinical Trial Registration:**

Registered December 9, 2016 at clinicaltrials.gov, First enrollment November 2019. ID NCT02987803, https://clinicaltrials.gov/ct2/show/NCT02987803

**Supplementary Information:**

The online version contains supplementary material available at 10.1186/s12884-022-05087-y.

## Background

Across the United States, hospital cesarean delivery rates vary dramatically, independent of demographic differences and pregnant people’s risks and preferences [1, 2]. While cesarean deliveries are often clinically necessary, up to 45% may be unindicated [3]. Compared to vaginal deliveries, cesarean deliveries are associated with three-fold higher rates of maternal complications and 50% higher costs [4–7]. Over three-quarters of pregnant people would prefer to not have unindicated cesarean deliveries, and an individual’s likelihood of a cesarean section is associated with the choice of hospital at which they deliver [8, 9].

To help patients select hospitals with lower cesarean rates and thereby lower the likelihood that they undergo cesarean sections, many states and consumer advocates such as The Leapfrog Group, Consumer Reports, and U.S. News and World Report have begun to publicly report hospital-level cesarean delivery rates [10–16]. However, few patients know where to access or seek out these data [17]. Furthermore, many prioritize the selection of an obstetrician or midwife over selection of a hospital and believe that a hospital’s cesarean delivery rate will not impact the care they receive [8, 17, 18].

In a pragmatic randomized controlled trial, we tested the hypothesis that an intervention that explained why hospital cesarean rates are important, simplified the presentation of these data, and made these data easier to access would lead more pregnant patients to select lower cesarean rate hospitals [19, 20]. Our intervention consisted of providing educational modules in a maternal health mobile app explaining the importance of a hospital’s cesarean delivery rate, translating hospital cesarean rate data into star ratings, using the language of “labor-friendly hospitals,” and providing an interactive tool that made it easier to find a hospital with a low cesarean rate.

## Methods

### Trial platform and recruitment

**Fig. 1:**
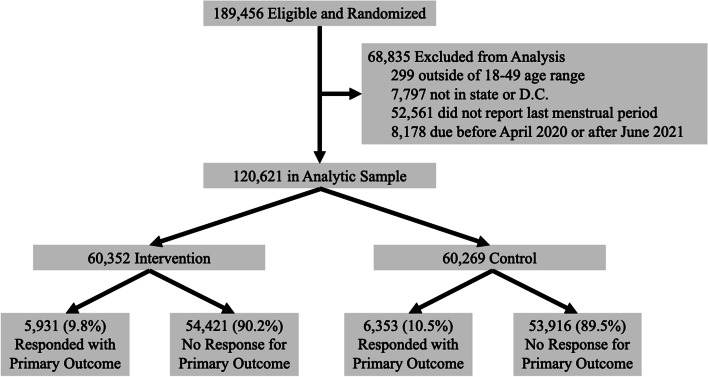
CONSORT Flow Diagram Note: Exclusion occurred after the randomization step in this pragmatic trial. Participants were immediately enrolled upon expressing interest. Exclusion criteria were self-reported by participants before randomization at their time of signing up for the app

We conducted this randomized controlled trial in 2019–2021 using the Ovia Fertility and Pregnancy mobile applications (apps). These apps, only available in English at the time of the study, offer a series of tools including articles customized to the patient and their interests (e.g., an article explaining the size of the fetus at the user’s stage of pregnancy) (Figure S1.1). These apps from Ovia Health predate the study and were available in most app stores. We conducted the study using the apps because of their large preexisting user base and because they could be easily modified to include new functionalities relevant to the study. To recruit participants, we presented a post in the “newsfeeds” of the app users who had been trying to conceive for fewer than five months or were 28–104 days into their pregnancies based on last menstrual periods. We included those still trying to conceive because we recognized that most of these people would soon become pregnant and sought to identify participants who had not yet chosen an obstetric provider. If a user expressed interest, they were immediately randomized 1:1 based on the last digit of their app user ID, which was automatically generated upon sign up for the app. Participants were excluded if they reported that they had already selected hospitals or obstetricians/midwives for their pregnancies because the intervention would likely have little impact in this population. We also excluded people if they were enrolled in two other Ovia programs (a health plan/employer program or a high-risk depression pregnancy intervention), as we did not want to interfere with those programs. We also excluded participants who did not report their last menstrual periods, had due dates outside the study period (April 2020-June 2021), reported ages outside of 18–49, or indicated they did not live in the United States. Trial recruitment and exclusions are outlined in the CONSORT diagram (Fig. 1).

### Intervention

Our intervention builds on the learnings of a prior pilot trial in which a similar intervention using informational tools increased participants’ familiarity with cesarean delivery rates but did not increase the likelihood of users selecting low-cesarean rate hospitals [21]. In the current trial, people in the intervention group were provided an interactive tool that showed hospital star ratings for the 10 closest hospitals within 50 miles of any zip code (star rating determinations explained below) as well as a series of educational modules on why it was important to select a hospital with a higher “labor-friendly” star rating. The interactive tool used a 5-star rating system instead of showing users the actual hospital cesarean rates because prior interviews suggested that many people had trouble translating a numerical rate into an actionable choice and prior research highlighted that simplifying quality data was key to increasing accessibility [22, 23]. We chose the term “labor-friendly” (paralleling the “baby friendly” designation) based on feedback from interviews that this phrase was appealing and accessible as well as evidence that hospitals with low cesarean section rates were more likely to provide additional support for labor (e.g., doulas) [24–26]. The 8 educational modules provided to the intervention population answered questions such as “Why does it matter if a hospital is labor-friendly?” and “What labor-friendly hospital is right for you?” with videos and articles.

People randomized to the control group were also given access to a hospital look-up tool as well as educational modules. The interactive hospital look-up tool provided to the control population was similar to the intervention tool but did not display any star ratings. The 2 control educational modules explained the different criteria someone could use to select a hospital and encouraged readers to go on hospital tours. (Screenshots of educational materials and tools for both arms available in supplemental materials).

Investigator blinding was less relevant in this study because researchers did not have any personal interaction with participants during the trial and all outcomes were self-reported by participants.

### Categorizing hospitals into star ratings based on cesarean rates

We used two data sources for hospital cesarean rates. For five states (Alabama, California, Massachusetts, Vermont, West Virginia), we used publicly reported primary cesarean rate data for all hospitals published by the state governments [12–16]. For all other states and D.C., which had no comparable public data, we used the self-reported nulliparous, term, singleton, vertex (NTSV) cesarean rates submitted by hospitals to the Leapfrog Group. Among known hospitals in these other states and D.C, 54.3% of hospitals did not submit data to Leapfrog. In Supplemental Materials, we detail dates of the data and differences in how the cesarean rate was measured based on data source (Table S1).

Within each state, we categorized hospitals into quintiles and assigned them star ratings based on their quintiles (e.g., 5 stars if the cesarean delivery rate was in the lowest quintile for the state). Hospitals for which cesarean rates were unavailable were marked “no data” and listed last on the interactive tool.

### Outcomes

Our primary outcome was the star rating of the delivery hospital each participant selected using the interactive hospital selection tool during pregnancy (hospital choice during pregnancy) (Figure S1.5 how participants entered these data). Participants were shown posts in their newsfeeds in the apps each week from the start of their enrollment asking them to report their chosen hospitals through the tool until they submitted choices. They were offered the opportunity to be entered into a $100 lottery for reporting.

Our secondary outcomes were survey questions focused on the importance of cesarean rate data and whether these data were used in the participants’ decisions (Table 1 provides the wording of questions). Starting in the second month after entry in the trial, participants had posts in their feeds asking them to fill out this survey. Again, participants were entered in a lottery for $100 for responding. If they responded to the survey more than once, we used their later responses.

**Table 1:** Participant survey responses regarding the importance of cesarean rates in selection of hospital

Survey Question	Response	Control	Intervention	*p*-value
Hospital Impact on Delivery *(Do you think the facility where you plan to deliver will impact your chance of having a C-section?)*		*n* = 4,064	*n* = 4,136	
	Very or somewhat likely	1,345 (33.1)	1,591 (38.5)	< 0.001
Use in Hospital Selection *(How much does the C-section rate of a facility matter to you when deciding where you'll deliver?)*		*n* = 4,340	*n* = 4,390	
	High or medium priority	3,226 (74.3)	3,343 (76.2)	0.05
Knowledge of Variation *(How different are healthcare facilities when it comes to quality of care?)*		*n* = 4,338	*n* = 4,390	
	Very or somewhat different	3,903 (90.0)	3,995 (91.0)	0.10

All users of the app (trial participants and non-participants) were asked to fill out a post-delivery survey that is sent to all app users regardless of whether they were part of this trial. Post-hoc, we added two secondary outcomes from this survey: hospital choice and self-reported delivery method (language of questions in supplemental materials). We linked each participant’s hospital choice to its star rating.

### Demographic and other data

Limited demographic data were collected by the Ovia apps, including enrollees’ ages and zip codes of residence. Using each participant’s zip code, we linked our dataset with data from the American Community Survey on median annual household income and the education levels of women ages 18–45 to estimate the incomes and education levels of the participants [27, 28]. Additionally, we used participant zip codes to determine which participants lived in urban areas as defined by the United States Census Bureau [29].

### Analysis

We compared the demographic characteristics of participants in the trial to those of pregnant people nationally using data from the Census and CDC [30–33]. We compared the star ratings of hospitals by conducting Welch’s *t*-tests, both for the primary outcome of choice during pregnancy and the secondary outcome of choice after pregnancy. We grouped Likert survey responses into binary categories and then used chi-squared tests to compare responses. We compared delivery methods between the control and intervention groups using a Pearson’s chi-squared test across all participants who indicated their delivery method as a post-hoc secondary outcome analysis.

In exploratory post-hoc subgroup analyses, we examined if the intervention’s impact was mediated by participant socioeconomic status (as measured by the median income in zip code), as we hypothesized that the impact of the intervention would be smaller among lower-income people given that other factors (e.g., insurance restrictions) may limit their hospital choices. We also conducted sub-group analyses to assess whether the intervention had a greater impact in communities with more choice of hospitals. Specifically, we stratified our analyses by whether the participant lived in a state that reported hospital cesarean rates and therefore fewer hospitals were missing star ratings, how many hospitals with a three star rating or higher were within 10 miles of the participant’s zip code, and whether there were hospitals with a difference in star ratings of at least two stars within 10 miles of the participant’s zip code. We calculated the star rating differences between the control and intervention arms in each subgroup. To determine if these differences were significant, we used linear regression models regressing on whether the participant was in the intervention or control, the sub-group (e.g., number of hospitals with a star rating of three or higher within 10 miles), and the interaction term between the two (variable of interest). All analyses were performed using R version 3.6.2.

The trial was approved by the Institutional Review Board of the Harvard T.H. Chan School of Public Health and registered at clinicaltrials.gov (Registration number NCT02987803, registered 09/12/2016). All participants consented through their agreements to the terms of use and privacy policy for the Ovia apps. A Data Safety Monitoring Board (DSMB) comprised of experts in the study content and statistical methods reviewed interim results in September 2020.

## Results

We enrolled 120,621 people in our trial (60,352 intervention and 60,269 control). Most participants were ages 25–34 (57.9%) and lived in urban zip codes (78.3%) (Table 2). Half (49.7%) of the participants resided in zip codes with median household incomes of less than $57,000. Sample characteristics were balanced across the control and intervention groups.

**Table 2:** Demographic Baseline Characteristics

Participant Characteristics	Control	Intervention	
	*n* = 60,269	*n* = 60,352	Standardized difference
	*n* (%)	*n* (%)	
Age			
18–24	18,444 (30.6)	18,341 (30.4)	0.0013
25–34	34,792 (57.8)	34,995 (58.0)	0.0015
35 +	7,033 (11.7)	7,016 (11.6)	0.0004
Region ^a^			
Midwest	12,588 (20.9)	12,662 (21.0)	0.0007
Northeast	9,422 (15.6)	9,652 (16.0)	0.0028
South	24,741 (41.0)	24,338 (40.4)	0.0042
West	13,601 (22.5)	13,617 (22.6)	0.0000
Median household income in zip code ^b^			
< $25,000	1,051 (2.5)	1,089 (2.6)	0.0013
$25,000—$49,999	13,535 (32.6)	13,301 (32.1)	0.0029
$50,000—$74,999	16,465 (39.6)	16,316 (39.4)	0.0018
$75,000—$99,999	6,896 (16.6)	7,033 (17.0)	0.0019
> $100,000	3,587 (8.6)	3,656 (8.8)	0.0013
Proportion with Bachelor's degree in zip code ^c^			
< 20%	11,375 (27.3)	11,149 (26.9)	0.0030
20%—< 30%	10,730 (25.8)	10,527 (25.4)	0.0027
30%—< 50%	12,849 (30.9)	13,137 (31.7)	0.0031
≥ 50%	6,637 (16.0)	6,628 (16.0)	0.0003
Rural or urban county ^d^			
Urban	43,321 (78.5)	43,097 (78.1)	0.0030
Rural	11,898 (21.5)	12,084 (21.9)	0.0020
Enrollment App			
Fertility	11,922 (19.8)	11,984 (19.9)	0.0005
Pregnancy	48,347 (80.2)	48,368 (80.1)	0.0005

Notes:

(a) Regions as listed in https://www.nationalgeographic.org/maps/united-states-regions/

(b) Source: U.S. Census American Community Survey, 2015–2019 5-year estimates from https://data.census.gov/cedsci. Median income in past 12 months by ZCTA, Table S1903. Data missing for 31.2% of participants due to low zip code population or incorrect zip code entry

(c) Source: U.S. Census American Community Survey, 2015–2019 5-year estimates from https://data.census.gov/cedsci. Educational attainment by ZCTA, Table S1501. Data missing for 31.1% of participants due to low zip code population or incorrect zip code entry

(d) Source: U.S. Census 2010 Urban Area to ZCTA Relationship File. Data missing for 8.4% of participants due to incorrect zip code entry

Compared to the national population of people giving birth, our study sample was slightly younger and more likely to reside in lower income and rural areas (Table S2).

### Choice of hospital during pregnancy

Among people enrolled in the trial, 10.2% (9.8% intervention, 10.5% control) reported their choices of hospitals during pregnancy. The average gestational age was 12 weeks at the time they provided this information. Among those who reported a hospital choice, 7.9% updated their hospital selection at least once. Compared to those who did not report this outcome, participants who reported this outcome lived in higher income communities (27.0% vs. 25.5% lived in zip codes with median incomes higher than $75,000) and were more likely to live in urban areas (84.1% vs. 77.6% in urban areas) (Table S3).

**Fig. 2:**
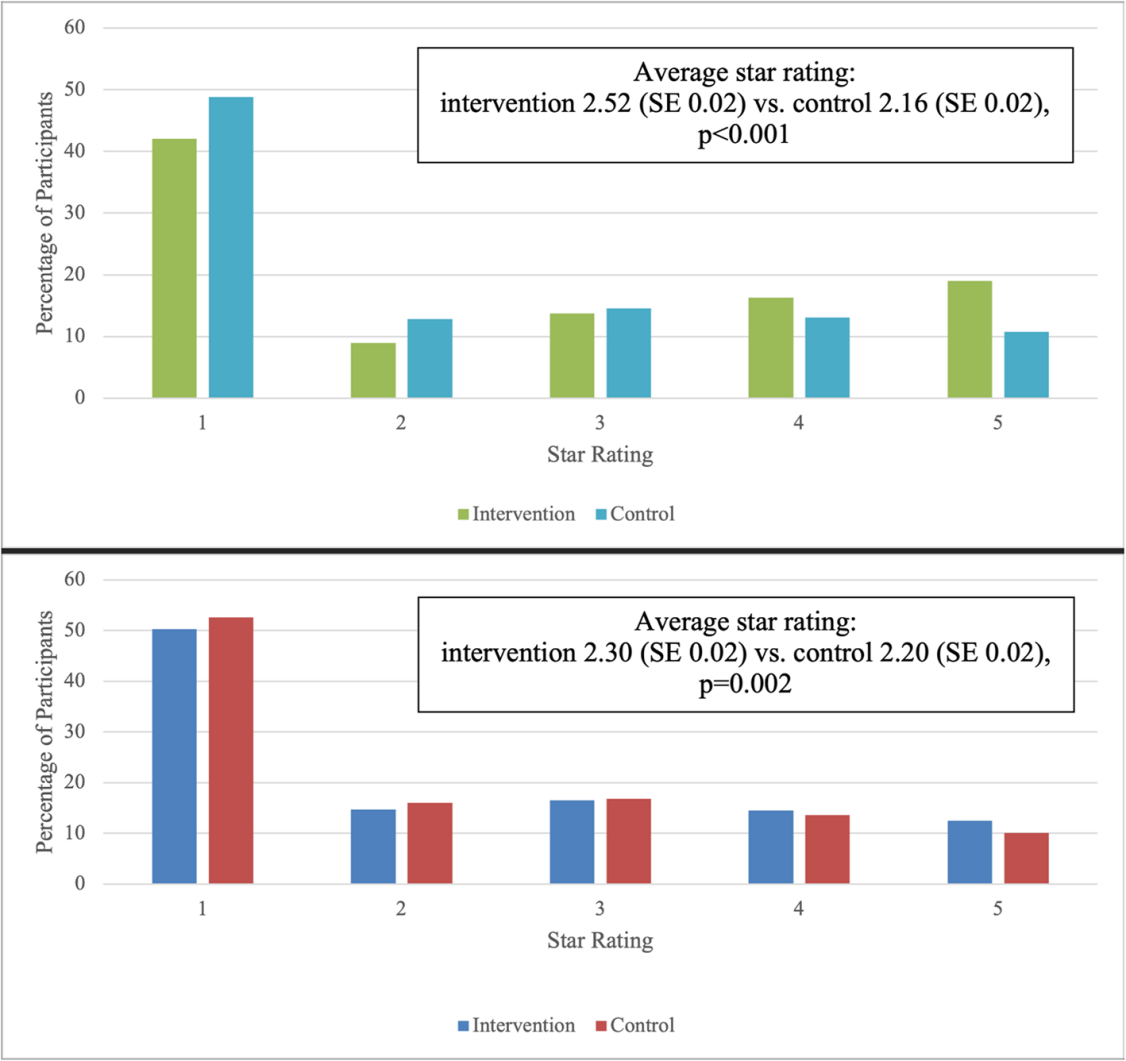
Cesarean-rate star ratings of hospitals selected by participants who reported hospital choices **A**. Choice of hospital reported during pregnancy (*n* = 5,931 intervention, *n* = 6,353 controls) **B**. Choice of hospital reported after delivery (*n* = 3,703 intervention, *n* = 3,808 controls) *Hospitals were assigned star ratings based on their cesarean rates, with higher star ratings assigned to hospitals with lower cesarean rates. **If participants selected hospitals without star ratings, their hospitals were treated as one-star for the purposes of analysis

The average star ratings of the hospitals selected by people in the intervention population were significantly higher than in controls (average star rating 2.61 (SD 1.60) intervention vs. 2.24 (SD 1.44), *p* < 0.001) (Fig. 2A). Of the participants who reported their hospitals, 19.0% of the intervention group selected 5-star hospitals while 10.8% of the control group selected 5-star hospitals.

### Choice of hospital reported after delivery

Among participants, 8,035 (6.7%; 6.6% intervention, 6.7% control) reported the hospitals in which they gave birth after their deliveries.

The average star rating of the hospitals where the intervention population gave birth was significantly higher than that of the control population (average star rating 2.30 (SD 1.42) intervention vs. 2.19 (SD 1.42) control, *p* = 0.001) (Fig. 2B). Of the participants who reported their hospitals, 11.5% of the intervention group selected 5-star hospitals while 9.2% of the control group selected 5-star hospitals.

Among the 1,681 (1.4%) of participants who reported their hospital choices both during pregnancy and after delivery, 60.5% and 54.5% reported the same hospitals at both instances for the control and intervention groups, respectively (Table S4).

### Secondary outcomes

People in the intervention group were more likely to believe that the choice of hospital impacts the likelihood of having a cesarean delivery (38.5% vs. 33.1%, *p* < 0.001, response rates of 6.9% and 6.7%). However, there were no differences in respondents’ beliefs that hospitals in their communities had different care quality levels (91.0% vs. 90.0%, *p* = 0.10, response rates of 7.3% and 7.2%) or that cesarean delivery rates are important to consider when choosing a hospital (76.2% vs. 74.3%, *p* = 0.050, response rates of 7.3% and 7.2%) (Table 1).

After delivery, 18,066 (29.9%) people in the intervention group and 18,139 (30.0%) people in the control group reported how their babies were delivered. There was no difference in the fraction of participants reporting they had cesarean deliveries (31.4% intervention, 31.4% control, *p* = 0.98).

### Sub-group analysis

Among the participants randomized to the intervention, 50,611 (83.9%) did not open any educational modules offered as part of the intervention and 50,241 (83.4%) did not use the hospital-look up tool. Among those in the intervention group who reported a delivery hospital, 3,419 (57.8%) did not open any educational modules. There were no clear differences in the mean star ratings of hospitals chosen during pregnancy stratified by the number of educational modules opened (0 modules 2.62, 1 module 2.62, 2 modules 2.65, 3 + modules 2.54) (Table S5).

**Fig. 3:**
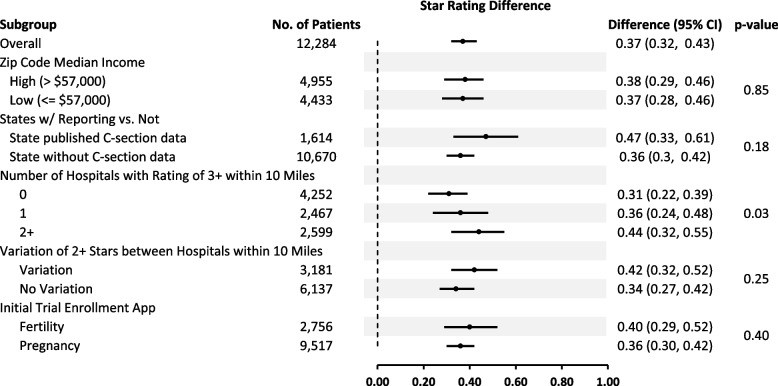
Difference in Average Star Ratings for Hospitals Selected by Intervention and Controls During Pregnancy Subgroup analyses *p*-values are based on the interaction term between the subgroup and the intervention in a multiple regression

We conducted exploratory analyses on the differential impact of the intervention across several subgroups (Fig. 3). We hypothesized that participants with more resources and higher education would be more likely to respond to the intervention. Using median income of the zip code of residence as a proxy for resources and education, we did not observe any substantive difference in the impact of the intervention between participants who lived in higher and lower education zip codes (*p* = 0.85).

Many hospitals did not report their cesarean rates, and therefore some participants had limited choice of hospitals with a star rating when they used the hospital look-up tool. This was less of an issue in the five states where we used state government data because hospitals were mandated to respond. The star rating differences between intervention and control groups were 0.47 stars for participants living in these five states vs. 0.36 stars for the rest of the nation (test of interaction, *p* = 0.18).

To further understand the influence of hospital choice, we examined the relationship between the number of hospitals with a 3-star or higher rating nearby and the impact of the intervention (0.31 difference in star ratings between intervention and controls among participants with no hospitals with a 3 star or higher rating within 10 miles, 0.36 for one hospital, 0.44 for more than one hospital, test of interaction *p* = 0.03). Relatedly, we compared participants who had a choice between hospitals with disparate star ratings (0.42 difference in star ratings between intervention and controls among participants where the maximum star rating is at least two stars greater than the minimum star rating among hospitals within 10 miles of the participant, 0.34 if this is not true, *p* = 0.25).

Finally, we wanted to see if the timing and context of patient recruitment relative to pregnancy made a difference in the effect of the intervention. We compared people who were recruited while they were trying to become pregnant and therefore using the fertility app (0.40 difference in star ratings between intervention and controls among participants recruited through the fertility app) vs. already pregnant (0.36 difference if recruited through the pregnancy app, *p* = 0.40).

## Discussion

### Principal findings

This randomized trial was motivated by the hypothesis that making cesarean delivery data more interpretable and accessible would encourage and enable pregnant people to use these data in the selection of hospitals to deliver their babies. Participants subject to the intervention were more likely to believe that cesarean rates were important in choosing a hospital and were more likely to select a hospital with a relatively low cesarean rate. However, there was no difference in participant likelihoods of having cesarean deliveries.

Our findings help inform the scientific literature on whether and how patients use publicly reported quality data to select a provider. With some notable exceptions in choices of nursing homes or health plans, prior reviews have highlighted that few patients are aware of publicly-reported quality data, and these data rarely impact provider selections [34–37]. Our results support prior laboratory studies that patients are more likely to use quality data to inform their provider choices if the data are more interpretable (e.g., star ratings, simplified presentations, patient-friendly language) and if they understand how the information can impact their own care [23].

### Clinical implications

The findings inform ongoing efforts to publicly report cesarean rate data, which are motivated by the goal of empowering pregnant people to make more informed delivery hospital selections. Our findings argue that simplifying data presentation by using star ratings and more patient-friendly terminology increases usability and therefore the impact of these data. Because the intervention encompassed many different components, we do not know which component was most important.

It is also important to emphasize that despite the inclusion of the educational program in the intervention explaining that the choice of a low-cesarean rate hospital could reduce their personal risks of delivery by cesarean, most participants in the intervention arm still did not believe this to be true. This belief will be a substantial barrier to influencing people’s delivery hospital choices that public reporting efforts alone cannot solve.

Additionally, despite a shift in hospital choices, we did not observe any difference in self-reported cesarean rates. This does not support the assumption underlying our study, and the rationale for public reporting of cesarean section data by states and other groups in general, that if more people shift to lower cesarean-rate hospitals, the overall cesarean rates in the population will fall.

### Research implications

Although there was a difference in the star ratings of hospitals selected during pregnancy (on average chosen in the first trimester), this difference was only modest, and the difference in star ratings of hospitals actually used for delivery was smaller. This shift between hospital choice early in pregnancy and actual delivery might be attributable to constraints based on proximity of the hospital or insurance. Further, only a minority of those randomized to the intervention engaged with the educational modules or hospital look up tool. Future research is needed to understand how to increase engagement in hospital cesarean rate data and what are the key barriers in using this information.

### Strengths and limitations

This pragmatic clinical trial was able to recruit a large and diverse national study population of over 100,000 participants using a mobile app. The intervention included a simple, low-cost, innovative tool that gives pregnant people readily comprehensible and actionable information. The intervention can be implemented relatively easily.

However, our results should be interpreted in the context of several key limitations. First, response rates were low for all outcomes, particularly survey results, and we reported outcomes on only those who responded. Second, the hospital data we used to create star ratings had limitations: the Leapfrog Group data lacked ratings for many hospitals, and the data were several years old at the time of the trial. However, when we limited the population to those states with more complete data, the effect of the intervention was similar. Third, many participants had limited hospital choices and therefore could not act on the data provided in the intervention [39]. Many hospitals had no data, more than a third of participants did not live within ten miles of a rated hospital, and almost half of participants did not live in zip codes within 10 miles of a hospital with a 3-star rating or higher. Fourth, we had limited demographic data, so we are unsure of the representativeness of our study population across many categories, particularly race. The absence of race/ethnicity data is particularly notable given the substantial racial inequities in maternal health [40]. Fifth, we do not have clinical data to determine for which births in the study population a cesarean delivery was indicated. We also did not ask participants whether they electively chose to have cesarean sections, but this is quite rare (< 1%) and therefore unlikely to drive our findings [41]. The study was conducted in part during the COVID-19 pandemic, and it is unclear how the pandemic changed patients’ delivery plans [38]. Additionally, we recognize that patient hospital choice is driven by a myriad of other considerations, including logistics, distance, continuity of care, and access to specialized services [42]. In this context, hospital cesarean section rate data can only have a limited impact. Finally, the intervention combined both educational modules and a selection tool, and we do not know which of the components drove the differences observed.

### Conclusions

In this randomized controlled trial of a large and diverse national study population, an intervention composed of educational modules, translations of hospital cesarean rates into patient-friendly star ratings, and an interactive tool to help select a delivery hospital increased the likelihood that people would plan to deliver at hospitals with lower cesarean rates. However, there was no change in participants’ likelihood of having a cesarean delivery.

## Supplementary Information

Additional file 1:

## Data Availability

All deidentified individual participant data analyzed for this manuscript along with data dictionaries, a statistical analysis plan, and analytic code may be available upon request. After publication, researchers may email the corresponding author. Valid requests may be fulfilled through online secure file transfer methods.
