# Supplementary material for: Impact of an educational program and decision tool on choice of maternity hospital: the delivery decisions randomized clinical trial
Source: BMC Pregnancy Childbirth. 2022 Oct 10;22:759. doi: 10.1186/s12884-022-05087-y (PMC9549827; doi:10.1186/s12884-022-05087-y)
Supplement: Supplementary file 1 — Additional file 1: [file 12884_2022_5087_MOESM1_ESM.docx]

**Appendix Table 1**: Cesarean Rate Data Sources

| Source | Year | Rate Calculation |
| --- | --- | --- |
| Alabama^a^ | 2015 | Primary Cesarean Birth Rate |
| California^b^ | 2016 | Primary Cesarean Birth Rate |
| Massachusetts^c^ | 2015 | Primary Cesarean Birth Rate |
| Vermont^d^ | 2015 | Primary Cesarean Birth Rate |
| West Virginia^e^ | 2014 | Primary Cesarean Birth Rate |
| Leapfrog^f^ | 2017 | NTSV Cesarean Birth Rate^g^ |

1. Alabama Center for Health Statistics. Alabama Vital Statistics: 2015, Table 5: Births by methods of delivery and hospital of occurrence with cesarean section and vaginal birth after cesearen rates Alabama, 2015. https://www.alabamapublichealth.gov/healthstats/assets/AVS2015.pdf
2. California Maternal Quality Care Collaborative. California Maternal Data Center. 2017. <https://www.cmqcc.org/maternal-data-center/california-mdc>. Note: These data were formerly accessible free of charge but are currently behind a paywall.
3. Massachusetts Department of Public Health. Massachusetts Births 2015, Table 18: Cesarean Deliveries and Vaginal Births after Cesarean (VBACs) by Licensed Maternity Facility, All Mothers, Massachusetts: 2015. https://www.mass.gov/doc/2015-birth-report-0
4. Vermont Department of Health. Vermont 2015 Vital Statistics, Table B-18: Type of Delivery by Hospital of Birth. <https://www.healthvermont.gov/sites/default/files/documents/pdf/Vital%20Statistics%20Bulletin%202015.pdf>
5. West Virginia Department of Health and Human Resources. West Virginia Vital Statistics 2014, Table 11: Births by Method of Delivery by Facility of Occurrence West Virginia, 2014. <http://www.wvdhhr.org/bph/hsc/pubs/vital/2014/2014Vital.pdf>
6. Not sure how to cite this. Not loading right now.
7. The Nulliparous, Term, Singleton, Vertex (NTSV) cesarean birth rate identifies the proportion of live babies born at or beyond 37.0 weeks gestation to women in their first pregnancy, that are singleton (no twins or beyond) and in the vertex presentation (no breech or transverse positions). The US Department of Health and Human Services in its Healthy People 2020 project simplified the name for non-obstetric audiences as “Low Risk Cesarean Birth among First Time Pregnant Women.” Source: https://www.cmqcc.org/focus-areas/quality-improvement/ntsv-c-sections

**Appendix Table 2**. Comparison of demographics of trial participants and national sample of births in the United States

| Population Characteristics | Trial^b^ | US^a,c,d^ |
| --- | --- | --- |
|  | n = 120,621 | % |
| Age ^a^ (available for 100% of trial population) |  |  |
| 18-24 | 30.5 | 22.3 |
| 25-34 | 57.9 | 57.8 |
| 35-49 | 11.6 | 18.7 |
| Region ^a^ (available for 100% of trial population) | | |
| Midwest | 20.9 | 22.1 |
| Northeast | 15.8 | 16.0 |
| South | 40.7 | 38.2 |
| West | 22.6 | 23.7 |
| Median household income in zip code ^b^ (available for 68.8% of trial population) | | |
| ≤ $25,000 | 2.6 | 16.1 |
| $25,000 - $50,000 | 32.4 | 20.8 |
| $50,000 - $100,000 | 39.5 | 31.9 |
| $100,000 - $150,000 | 16.8 | 16.9 |
| > $150,000 | 8.7 | 14.2 |
| Rural or urban county ^c, d^  (available for 91.6% of trial population) | | |
| Urban | 78.3 | 81.5 |
| Rural | 21.7 | 18.5 |

1. Martin JA, Hamilton BE, Osterman MJK, Driscoll AKD. Births: Final Data for 2019. *National Vital Statistics Reports.* 2021;70(2):1-51. <https://dx.doi.org/10.15620/cdc:100472>
2. The trial numbers are based on zip code median incomes for participants while the US numbers are based on individual-level data from pregnant people. Source: U.S. Census. Fertility of Women in the United States: 2018, Table 7: Household Income and Income per Household Member Among Women with a Birth in the Past Year, by Marital Status: 2017. https://www.census.gov/data/tables/2018/demo/fertility/women-fertility.html
3. Ely DM, Hamilton BE. Trends in fertility and mother’s age at first birth among rural and metropolitan counties: United States, 2007–2017. *NCHS Data Brief*. 2018;323:1-8. https://www.cdc.gov/nchs/products/databriefs/db323.htm
4. Ingram DD, Franco SJ. 2013 NCHS urban–rural classification scheme for counties. *Vital Health Stat.* 2014;2(166):1-73. https://www.cdc.gov/nchs/data/series/sr_02/sr02_166.pdf

**Appendix Table 3**. Comparison of participant demographics by whether they reported the primary outcome (reporting choice of delivery hospital during pregnancy)

| Participant Characteristics | Response | | No Response | |
| --- | --- | --- | --- | --- |
|  | n = 12,284 | | n = 111,740 | |
|  | Control  n=6,353 | Intervention  n=5,931 | Control  n=53,916 | Intervention  n=54,421 |
|  | n (%) | n (%) | n (%) | n (%) |
| Age |  |  |  |  |
| 18-24 | 1,726 (27.2) | 1,591 (26.8) | 16,718 (31.0) | 16,750 (30.8) |
| 25-34 | 3,830 (60.3) | 3,646 (61.5) | 30,962 (57.4) | 31,349 (57.6) |
| 35+ | 797 (12.5) | 694 (11.7) | 6,236 (11.6) | 6,322 (11.6) |
| Region ^a^ |  |  |  |  |
| Midwest | 1,377 (21.7) | 1,282 (21.6) | 11,285 (20.9) | 11,306 (20.8) |
| Northeast | 1,113 (17.5) | 984 (16.6) | 8,539 (15.8) | 8,438 (15.5) |
| South | 2,481 (39.1) | 2,320 (39.1) | 21,857 (40.5) | 22,421 (41.2) |
| West | 1,382 (21.8) | 1,345 (22.7) | 12,235 (22.7) | 12,256 (22.5) |
| Median household income in zip code ^b^ | |  |  |  |
| < $25,000 | 95 (2.0) | 77 (1.7) | 994 (2.7) | 974 (2.6) |
| $25,000 - $49,999 | 1,495 (31.0) | 1,367 (29.9) | 11,806 (32.3) | 12,168 (32.9) |
| $50,000 - $74,999 | 1,953 (40.6) | 1,870 (40.9) | 14,363 (39.3) | 14,595 (39.5) |
| $75,000 - $99,999 | 859 (17.8) | 847 (18.5) | 6,174 (16.9) | 6,049 (16.4) |
| > $100,000 | 413 (8.6) | 411 (9.0) | 3,243 (8.9) | 3,176 (8.6) |
| Proportion with Bachelor's degree in zip code ^c^ | | |  |  |
| < 20% | 1,267 (26.3) | 1,139 (24.9) | 9,882 (27.0) | 10,236 (27.7) |
| 20% - <30% | 1,208 (25.1) | 1,159 (25.3) | 9,319 (25.4) | 9,571 (25.9) |
| 30% - <50% | 1,615 (33.5) | 1,512 (33.0) | 11,522 (31.5) | 11,337 (30.6) |
| ≥ 50% | 732 (15.2) | 767 (16.8) | 5,896 (16.1) | 5,870 (15.9) |
| Rural or urban county ^d^ |  |  |  |  |
| Urban | 4,952 (83.4) | 4,701 (84.8) | 38,145 (77.5) | 38,620 (77.7) |
| Rural | 986 (16.6) | 845 (15.2) | 11,098 (22.5) | 11,053 (22.3) |

Notes:
(a) Regions as listed in <https://www.nationalgeographic.org/maps/united-states-regions/>.
(b) Source: U.S. Census American Community Survey, 2015-2019 5-year estimates from <https://data.census.gov/cedsci>. Median income in past 12 months by ZCTA, Table S1903. Data missing for 31.2% of participants due to low zip code population or incorrect zip code entry.
(c) Source: U.S. Census American Community Survey, 2015-2019 5-year estimates from <https://data.census.gov/cedsci>. Educational attainment by ZCTA, Table S1501. Data missing for 31.1% of participants due to low zip code population or incorrect zip code entry.
(d) Source: U.S. Census 2010 Urban Area to ZCTA Relationship File. Data missing for 8.5% of participants due to incorrect zip code entry.

**Appendix Table 4:** Consistency of Hospital Choices During and After Delivery for Participants who Reported a Hospital in Both Periods, n=1681

|  | Control (n=910)  n (%) | Intervention (n=771)  n (%) |
| --- | --- | --- |
| Consistent (Hospital selected at both time points was the same) | 551 (60.5) | 420 (54.5) |
| Inconsistent (Hospital selected at the two points was different) | 359 (39.5) | 351 (45.5) |

**Appendix Table 5:** Mean Hospital Star Ratings by Engagement Levels

Table 5a: Mean Hospital Star Ratings by Engagement Levels in the Intervention Group

| Number of Educational Modules in Program Opened during Study | n (%) of intervention population who reported their delivery hospital during pregnancy | Mean Star Rating of Hospital Chosen |
| --- | --- | --- |
| 0 | 3,430 (57.8) | 2.62 |
| 1 | 1,167 (19.7) | 2.62 |
| 2 | 676 (11.4) | 2.65 |
| 3+ | 658 (11.1) | 2.54 |

Table 5b: Mean Hospital Star Ratings by Engagement Levels in the Control Group

| Number of Educational Modules in Program Opened during Study | n (%) of intervention population who reported their delivery hospital during pregnancy | Mean Star Rating of Hospital Chosen |
| --- | --- | --- |
| 0 | 5,906 (93.0) | 2.25 |
| 1+ | 447 (7.0) | 2.07 |

Figure S1

The photographs in these articles do not contain study participants. They were stock photos acquired by Ovia as a visual supplement to the apps and text of the articles.

1.1 Screenshots of Ovia Health Applications

Fertility:


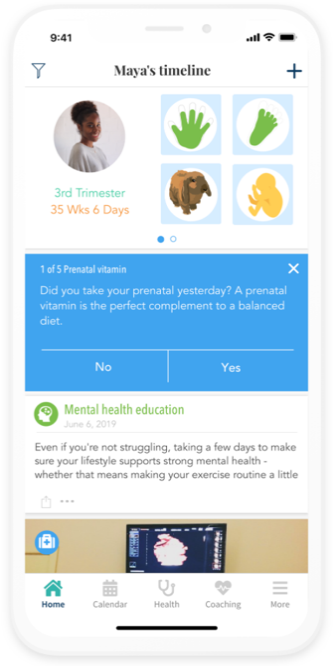

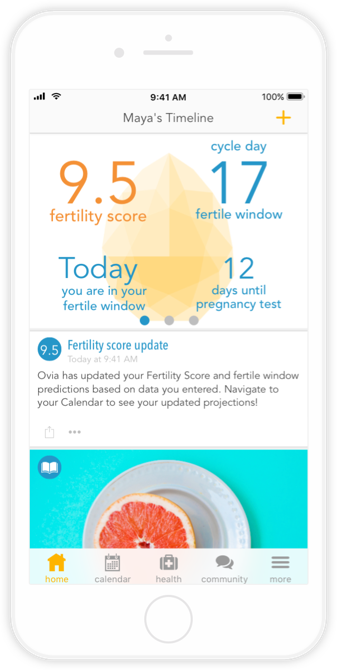


Pregnancy:


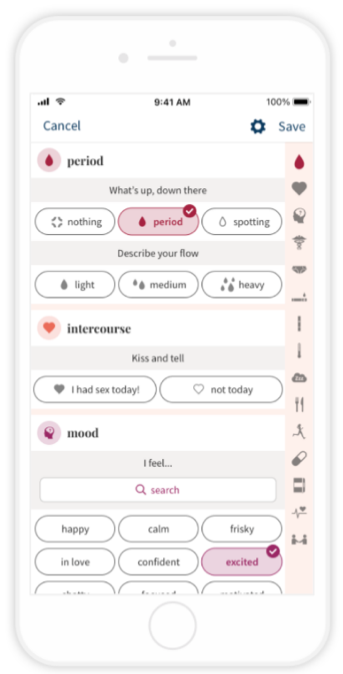

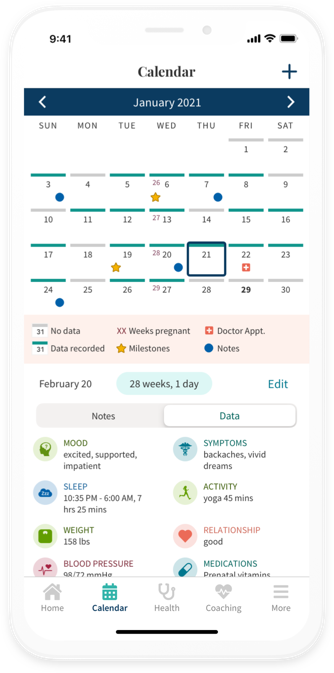


1.2 Text of Articles for the Intervention Group

Module list:

1. Why should you consider a labor-friendly hospital?
2. Why does it matter if a hospital is labor-friendly?
3. What labor-friendly hospital is right for you?
4. What does Ovia’s Hospital Lookup Tool do?
5. What should I look for in a hospital?
6. Who can help me decide on a delivery hospital?
7. How Ovia’s Hospital Lookup Tool can help you find a hospital
8. VIDEO: Delivery information every mom should know (in Ovia Pregnancy app only, not in Fertility app)

Example Article:

**
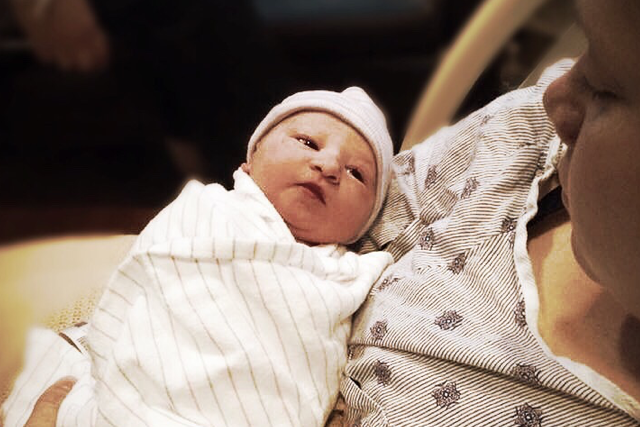
Why should you consider a labor-friendly hospital?**

Because of the way labor and delivery are depicted in movies and TV, you might think that women never choose their hospitals in advance — they just rush to whatever facility is closest when their water breaks at the top of the Empire State Building (and give birth after only minutes of pushing).

In reality, choosing a hospital is among the first and most important parts of developing a birth plan, and there's more to consider than just location. How will each hospital support you during early labor? Can you expect that your nurse will spend enough time with you in the room, monitoring your progress and coaching you as needed? How many babies at each hospital are delivered via C-section? Trying to understand answers to questions like these can tell you how "labor friendly" a hospital is, which could have a big impact on your birth experience.

Ovia defines a labor-friendly hospital as one that has a lower C-section rate, which we believe may indicate they:

- Consistently provides staffing and personal attention needed for support during labor
- Places an emphasis on helping patients follow their birth plans with the result of fewer risks and extended recovery from unnecessary C-sections

According to the official recommendation from ACOG, vaginal deliveries are the safer option for mom and baby, as medically unnecessary c-sections carry a great deal of risk and is linked to a longer recovery time.

When interventions like C-sections become necessary (and sometimes they are necessary), labor-friendly hospitals will make you aware of both necessity and risks of the interventions and allow you to contribute to the decision.

By allowing labor to progress as intervention-free as possible, labor-friendly hospitals reduce the number of medically unnecessary C-sections — and have lower overall C-section rates as a result. The lower the C-section rate, the more labor-friendly the hospital.

That means you can look up just how labor-friendly your local hospitals are.

When you’re choosing a hospital, it is important to try to learn as much as possible about the facility by researching their ratings and talking to friends and family who have given birth there or know someone who has, and look up their ratings. It’s not always possible to meet the doctor, midwife, or nurse who will be helping you through labor, but by choosing a labor-friendly hospital with Ovia’s Hospital Lookup Tool, you are taking all the right steps to receive high quality care.

Use Ovia's Hospital Lookup Tool to search for hospitals near you and see our ratings of which ones are the most labor-friendly.

[**Go to Hospital Lookup Tool**](oviapregnancy://native-health/program-detail/31)

1.3 Screenshots of Hospital Search Tool

Intervention:


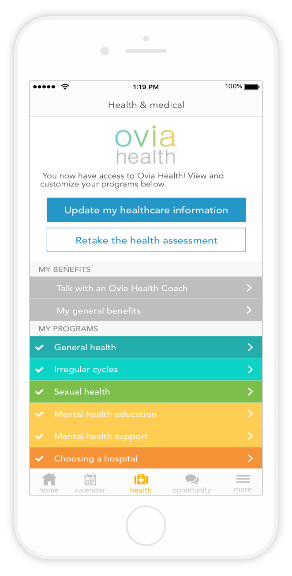

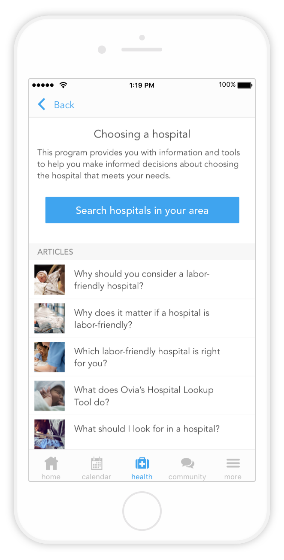

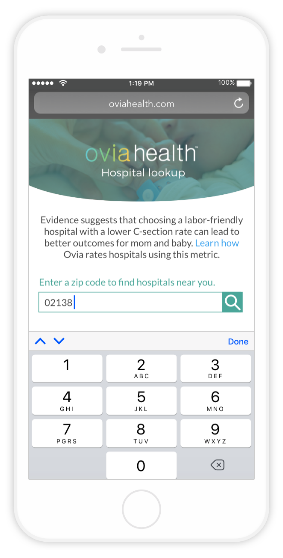

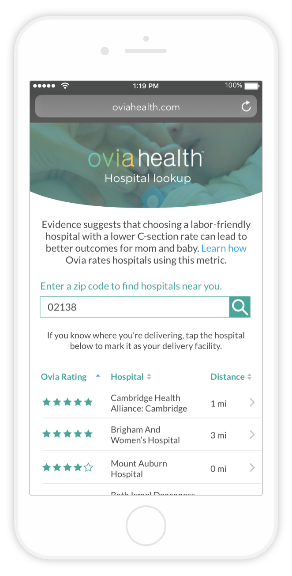

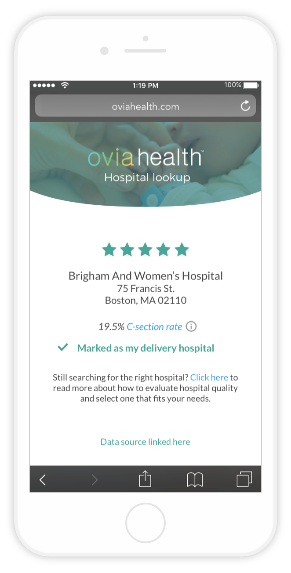


Control:


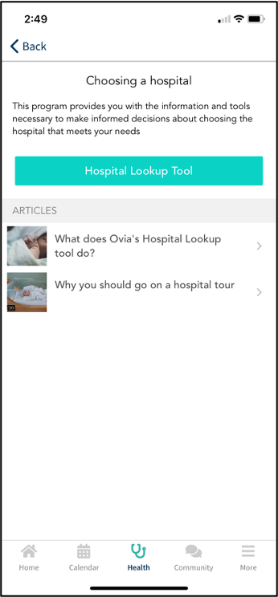

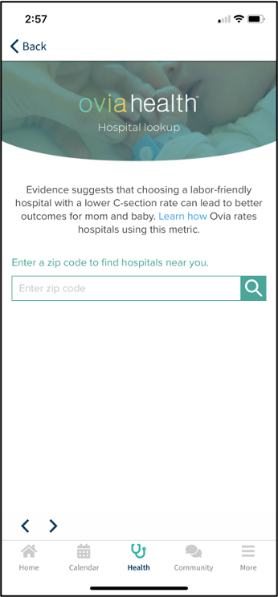

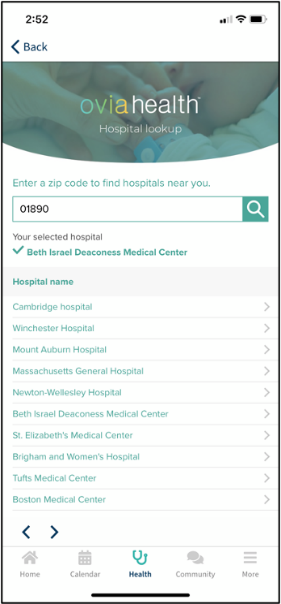

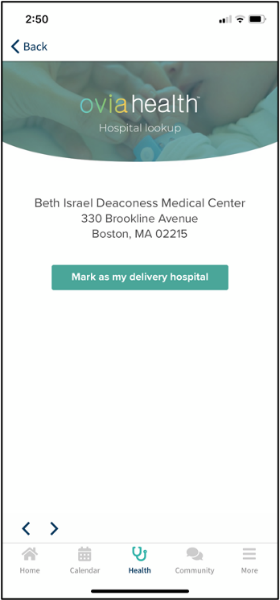

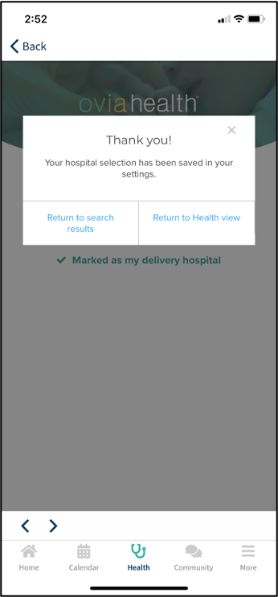


1.4 Text of Articles for the Control Group

Module List:

1. What does Ovia’s Hospital Lookup Tool do?
2. Why you should go on a hospital tour

Example Article Text:

**
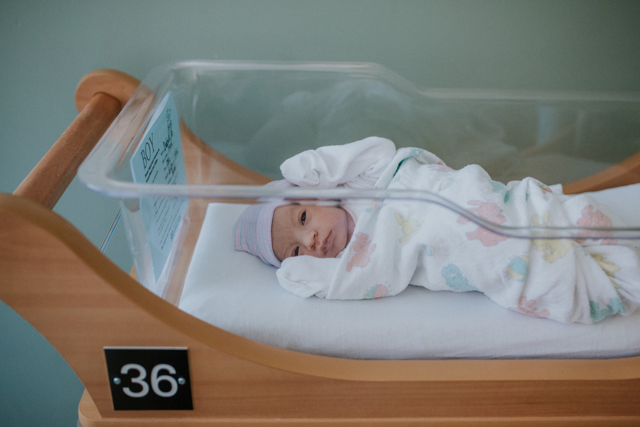
Why you should go on a hospital tour**

Throughout TTC and, eventually, pregnancy, you might see a lot of checklists and tips for what to pack in your hospital bag. However, most of those resources don't mention the most important part of your hospital bag: where you'll be opening it!

So much about your birth experience — including what you need to bring and what you can expect on the day of your delivery — depends on the hospital you choose. What’s the best way to find out what your delivery day will be like? A hospital tour!

Not only does a hospital tour allow you to familiarize yourself with the hospital's physical location and layout, it also gives you a chance to interact with the staff and ask questions. Touring a hospital, especially while you're still deciding what hospital is best for you, will help you understand and visualize your future birth experience. Take a hospital tour to:

- **Learn where to park** (a small piece of information that can provide peace of mind!)
- **Familiarize yourself with the layout** (including hunting down the best vending machines for your favorite snacks)
- **Get to know the staff**(most hospital tours are given by childbirth educators and/or nurses, and some hospitals will even do open houses with doctors)

Hospital tours are usually no longer than 30 minutes, and it's easy to make an appointment. Just call the hospital, find out what days are available, and reserve a spot. If you're not sure what hospitals you're interested in touring yet, use Ovia's Hospital Lookup Tool to find the right hospital near you!

[**Go to Hospital Lookup Tool**](oviafertility://native-health/program-detail/132)

1.5 Screenshots of Hospital Selection


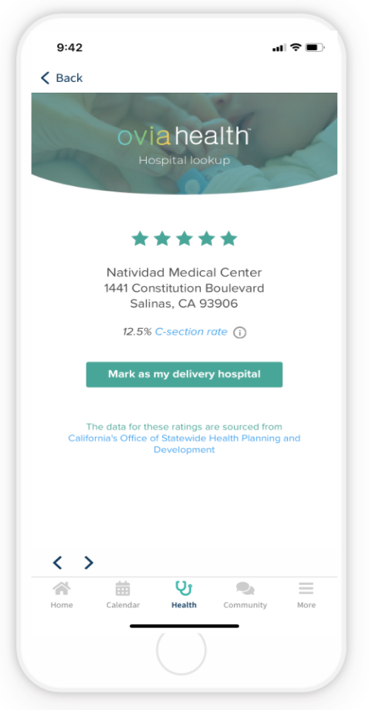


1.6 Screenshots of Surveys in Ovia


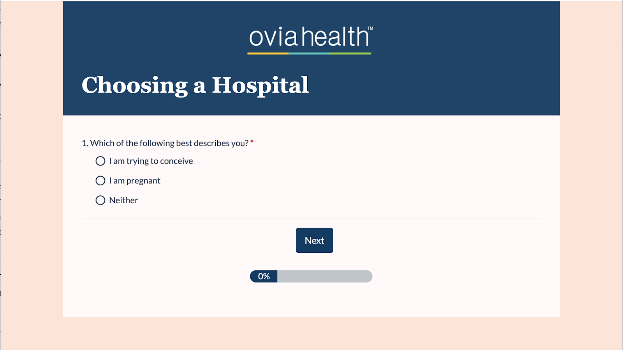

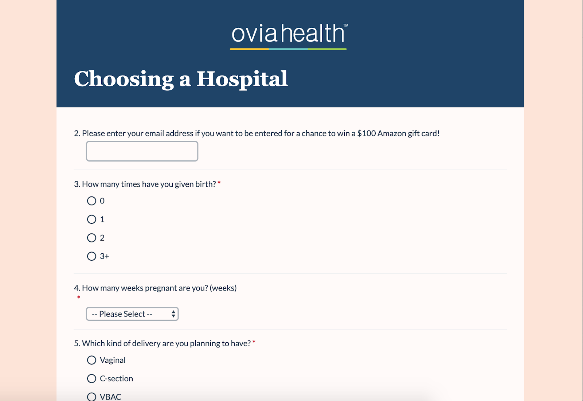


1.7 Screenshots of Birth reporting in Ovia
